# Bisphosphonate use in patients undergoing total knee arthroplasty reduces overall and aseptic revisions and periprosthetic bone mineral density loss: A systematic review from the FP‐UCBM Knee Study Group

**DOI:** 10.1002/ksa.12802

**Published:** 2025-07-18

**Authors:** Giancarlo Giurazza, Marco Edoardo Cardinale, Edoardo Franceschetti, Stefano Campi, Giuseppe Francesco Papalia, Pietro Gregori, Francesco Rosario Parisi, Augusto Ferrini, Michele Paciotti, Rocco Papalia

**Affiliations:** ^1^ Fondazione Policlinico Universitario Campus Bio‐Medico, Via Álvaro del Portillo Roma Italy; ^2^ Department of Medicine and Surgery Research Unit of Orthopaedic and Trauma Surgery, Università Campus Bio‐Medico di Roma, Via Álvaro del Portillo Roma Italy; ^3^ Oncological Orthopaedics Department IFO ‐ IRCCS Regina Elena National Cancer Institute, Via Elio Chianesi Rome Italy

**Keywords:** bisphosphonates, osteoporosis, periprosthetic bone mineral density (BMD) loss, revision TKA, total knee arthroplasty (TKA)

## Abstract

**Purpose:**

Bisphosphonates (BPs), widely used for osteoporosis management, have garnered attention in the context of total knee arthroplasty (TKA) for their potential to preserve periprosthetic bone mineral density (BMD) and mitigate risks such as aseptic loosening, periprosthetic fractures, and implant failure. This study systematically reviewed the current literature on the topic, hypothesising that BP therapy reduces the risk of postoperative adverse outcomes in patients undergoing TKA.

**Methods:**

A systematic literature search was conducted on 1 December 2024 using PubMed, Cochrane Library, Scopus and Google Scholar. Inclusion criteria were: English‐language randomised controlled trials (RCTs), comparative prospective or retrospective studies, and studies evaluating postoperative outcomes (including all‐cause and aseptic revisions, periprosthetic BMD loss, periprosthetic fractures and implant migration) in patients undergoing TKA who received BP treatment. Exclusion criteria were: computational studies; case reports; studies focusing uniquely on the surgical technique; studies reporting only cumulative data for total joint arthroplasty.

**Results:**

A total of 14 studies (480,294 patients) were included. Six studies focused exclusively on osteoporotic patients, six on non‐osteoporotic patients and two on a mixed BMD population. The rate of all‐cause and aseptic revisions was 1.5% and 1.1% respectively for BP users, and 2.3% and 2.5%, for non‐BP users. Mean pre‐/post‐operative variation in BMD was –0.04 for BP users and –0.2 for non‐BP users. The risk of bias was graded as low using the ROB 2 tool for RCTs and the Newcastle–Ottawa Scale for observational studies.

**Conclusion:**

Bisphosphonate use in patients undergoing total knee arthroplasty is associated with lower rates of overall and aseptic revisions, reduced periprosthetic BMD loss, and inconclusive effects on periprosthetic fractures and implant migration.

**Level of Evidence:**

Level IV.

**Abbreviations**
BMD: bone mineral density
BP: bisphosphonates
HR: hazard ratio
IT: intermediate term
LT: long term
OR: odds ratio
PPFx: periprosthetic fractures
ST: short term
TKA: total knee arthroplasty

## INTRODUCTION

Total knee arthroplasty (TKA) represents the gold standard for restoring mobility and relieving symptoms in patients with end‐stage knee osteoarthritis, offering excellent outcomes [10, 23, 51].

Osteoporotic patients undergoing TKA are exposed to higher risks of intra‐operative and post‐operative complications, including revision surgery [17], transfusions and hospital readmissions. It is estimated that approximately one quarter of TKA patients suffer from osteoporosis [49], though only about 20% receive any specific treatment, underscoring a significant gap in care [2, 27]. Bisphosphonates (BP), a cornerstone therapy for osteoporosis [5], are gaining clinical interest in this context for their potential role in slowing bone mineral density (BMD) loss around implants, thereby mitigating risks such as aseptic loosening, periprosthetic fractures, and implant failure [32, 36].

Despite emerging evidence supporting BP use in TKA patients, the existing literature lacks a comprehensive synthesis of its implications. This study aimed to address this gap through a systematic review of the current literature, with the hypothesis that BP treatment reduces the risk of post‐operative adverse outcomes in patients undergoing TKA.

## MATERIALS AND METHODS

This study adhered to the Preferred Reporting Items for Systematic Reviews and Meta‐Analysis (PRISMA) guidelines and utilised the PRISMA checklist [33].

### Eligibility criteria

English‐language randomised controlled trial (RCT), comparative prospective and retrospective studies evaluating postoperative outcomes (periprosthetic fractures, periprosthetic BMD loss, implant migration, aseptic and all‐cause revisions) in patients undergoing TKA who received BP treatment were considered eligible. There were no restrictions to the timing of BP administration. Computational studies, case reports, studies focusing uniquely on the surgical technique and studies reporting only cumulative data for total joint arthroplasty (combining TKA and THA without separating outcomes by procedure type) were excluded.

### Information sources and search strategy

**Figure 1:**
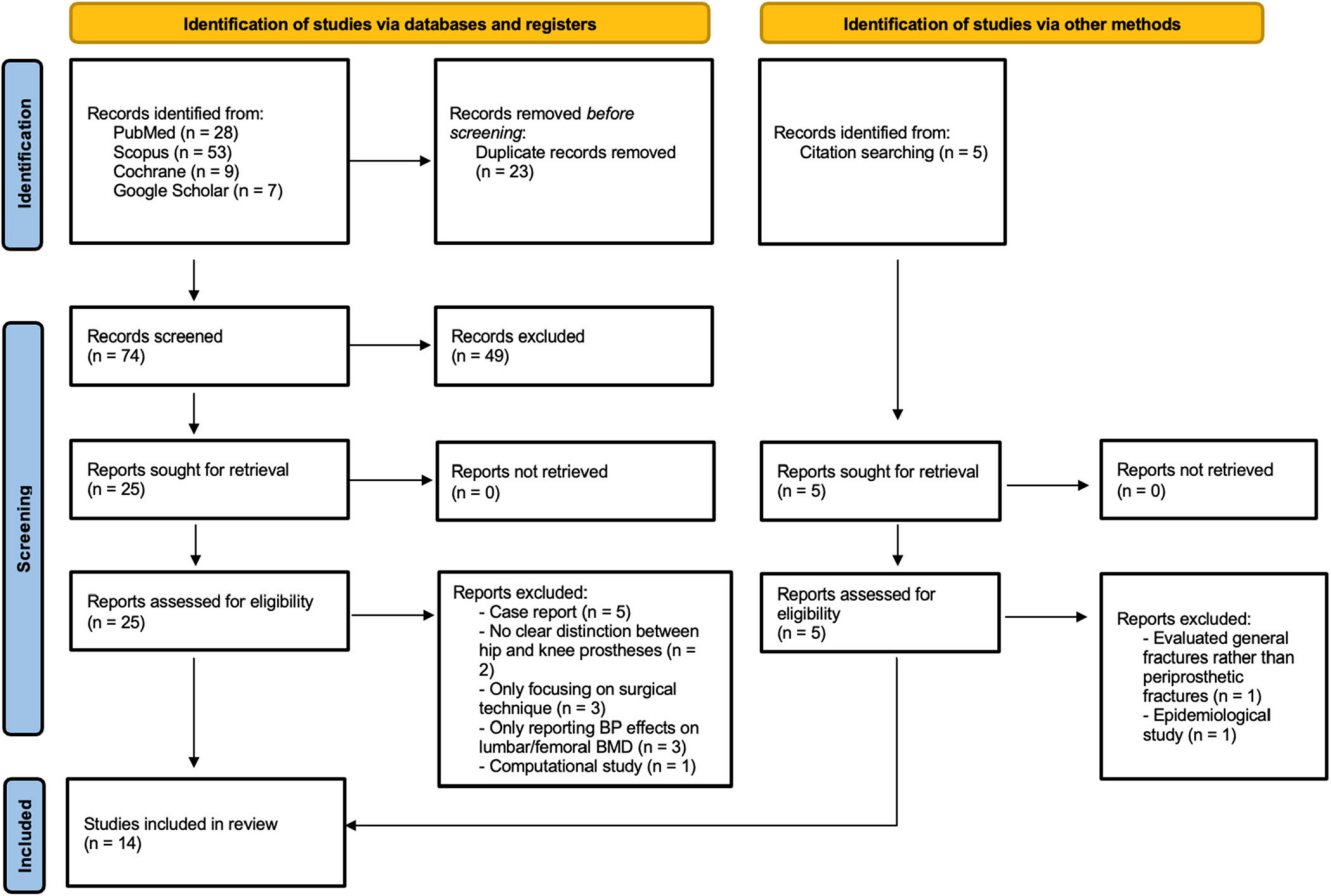
PRISMA 2020 flow diagram; PRISMA, Preferred Reporting Items for Systematic Reviews and Meta‐Analysis.

A systematic literature search was conducted from inception to 1 December 2024, using Pubmed Medline, Cochrane Library, Scopus and Google Scholar databases (PROSPERO registration number: CRD420251004575). The strings used for initial screening were: ((((TKA[Title/Abstract]) OR (total knee arthroplasty [Title/Abstract])) OR (TKR[Title/Abstract])) OR (total knee replacement [Title/Abstract])) AND (bisphosphonate [Title/Abstract]). Thus, 97 articles were identified by the initial literature search and 5 additional articles were identified through cross‐reference search. After removing duplicates, two authors (MEC and GG) independently screened the abstracts for relevance, followed by full‐text assessment. A total of 16 were excluded for the following reasons: case report (*n* = 5), no clear differentiation between hip and knee prostheses (*n* = 2), evaluated general fractures rather than periprosthetic fractures (*n* = 1), only focusing on surgical technique (*n* = 3), only reporting BP effects on lumbar/femoral BMD (*n* = 3), computational study (*n* = 1) and epidemiological study (*n* = 1). Ultimately, 14 articles met the inclusion criteria as illustrated in the PRISMA flow diagram [33] (Figure 1).

### Data collection

The following variables were extracted from the selected studies and subsequently summarised in tables using Excel: author name, year of publication, type of study, level of evidence, number of patients (divided into BP users and non‐BP users), sex distribution, mean age, prior diagnosis of osteoporosis, BP treatment protocol (specific molecule, dosage, duration, timing of administration), study results and conclusions. According to a widely used criterion [26, 32, 36], preoperative BP use was defined as continuous bisphosphonate therapy for a minimum of six months prior to surgery.

### Risk of bias assessment

The risk of bias of the included studies was independently assessed using two different tools according to study design: the ROB 2 tool [41] was applied to RCTs, while the Newcastle–Ottawa Scale (NOS) [48] was used for observational studies.

## RESULTS

The 14 included studies, encompassing a total of 480,294 patients, spanned from 2000 [21] to 2025 [25]. The total post‐TKA follow‐up period ranged from 1 [21, 25, 40] to 15 years [39]. When reported, the mean age was 69 ± 7.2 years. Based on timing of BP administration, studies were categorised in perioperative (both preoperative and postoperative, five studies), postoperative (eight studies) or intraoperative (one study). Further details regarding study type, level of evidence and demographic characteristics are summarised in Table 1. Study methods, results, and conclusions are summarised in Table 2.

**Table 1:** Selected studies, level of evidence and demographic characteristics.

Author	Year	Type of study	Level of evidence	Study population	Sex	Age (mean ± SD)	Osteoporosis	BP treatment (timing)		Implant fixation
Forlenza et al. [8]	2024	Retrospective registry study PearlDrive Database (USA)	III	42.116 pt (50% BP users, 50% non‐BP users)	21.058 female (50%) 21.058 male (50%)	71 ± 6.4	Yes	Pre‐operative	Post‐operative	Cemented
								Yes	Yes	
Hansson et al. [16]	2009	Randomised controlled study	I	60 pt (50% BP users, 50% non‐BP users)	35 female (58.3%) 25 male (41.7%)	68 (range 51–80)	No	No	Yes	Uncemented
Hilding et al. [21]	2000	Randomised controlled study	I	50 pt (50% BP users, 50% non‐BP users)	NR	Range 60–75	No	Yes	Yes	Cemented
Hilding et al. [20]	2006	Randomised controlled study	I	47 pt (48.9% BP users, 51.1% non‐BP users)	NR	Range 60–75	No	Yes	Yes	Cemented
Hilding et al. [18]	2007	Randomised controlled study	I	50 pt (50% BP users, 50% non‐BP users)	NR	Range 60–75	No	Intraoperative use		Cemented
Jaroma et al. [24]	2015	Randomised controlled study	I	26 pt (53.8% BP users, 46.2% non‐BP users)	17 female (65.4%) 11 male (34.6%)	67 ± 7	No	No	Yes	Cemented
Katz et al. [25]	2025	Retrospective registry study PearlDrive Database (USA)	III	24.162 pt (50% BP users, 50% non‐BP users)	23.892 female (97.8%) 270 male (2.2%)	70.96 ± 6.25	Yes	Yes	Yes	NR
Lee et al. [28]	2024	Retrospective registry study PearlDrive Database (USA)	III	26.984 pt (39.2% BP users, 60.8% non‐BP users)	23.152 female (85.8%) 3.832 male (14.2%)	69.3 ± 7.6	Yes	Yes	Yes	NR
Namba et al. [32]	2016	Retrospective registry study Kaiser Permanente Southern California Database (USA)	III	34.116 pt (19.6% BP users, 80.4% non‐BP users)	21.493 female (63%) 12.623 male (37%)	67.3 ± 9.1	9.2% osteoporotic, 30.5% osteopenic, 26.2% normal, 34.1% did not have a DEXA scan within 5 years	No	Yes^a^	90.1% cemented, 1.4% uncemented, 4.6% hybrid, 3.6% missing
Ro et al. [38]	2018	Retrospective registry study National Health Insurance Service Database (Korea)	III	331.660 pt (22.9% BP users, 77.1 non‐BP users)	291.860 female (88%) 39.800 male (12%)	68.8 ± 7	Osteoporosis in 23% of BP users and 8% of non‐BP users	No	Yes^b^	NR
Shih et al. [39]	2024	Retrospective registry study National Health Insurance Research Database (Taiwan)	III	20.920 pt (20% BP users, 80% non‐BP users)	16.506 female (68.9%) 4.414 male (21.1%)	68.8 ± 8.5	Yes	No	Yes	NR
Soininvaara et al. [40]	2002	Randomised controlled study	II	19 pt (42.1% BP users, 51.9% non‐BP users)	12 female (63.2%) 7 male (36.8%)	67 ± 8.6	Yes	No	Yes	Cemented
Ueyama et al. [44]	2020	Prospective study	II	30 pt (bilateral TKA, 100% BP users)	29 female (96.7%) 1 male (3.3%)	72.2 ± 6.4	Yes	No	Yes	Cemented
Wang et al. [47]	2006	Randomised controlled study	I	54 pt (53.7% BP users, 66.3% non‐BP users)	54 female (100%)	69.8 ± 5.9	No	No	Yes	Cemented

Abbreviations: BP, bisphosphonate; NR, not reported; TKA, total knee arthroplasty.

^a^ BP users defined as: Patients treated for ≥6 months before revision surgery or patients treated for ≥6 months without undergoing revision surgery.

^b^ BP users defined as patients who had received at least 1 WHO‐defined daily dose of oral BP before revision surgery.

**Table 2:** Objectives, results and conclusions of the included studies.

Author	BP regimen	Follow‐up^a^	Study results	Study conclusions
Forlenza et al. [8]	Peri‐op administration of bisphosphonates (starting minimum 6 months before surgery) Further details: NR	2 years	All cause revision rate: ‐ BP users: 1.8% ‐ non‐BP users: 1.5% (*p* = 0.022) Aseptic revision rate: ‐ BP users: 0.7% ‐ non‐BP users: 0.6% (*p* = 0.469) PPFx rate (All TKAs): ‐ BP users: 0.7% ‐ non‐BP users: 0.8% (*p* = 0.068) PPFx rate in cemented vs cementless TKA: ‐ BP users: 1.0 vs. 1.3%; *p* = 1 ‐ non‐BP users: 0.2% vs. 1.7%; *p* = 0.015	Peri‐op BP treatment reduces the risk of all‐cause revision reduces PPFx risk only in cementless, but not reduce the risk of aseptic revision
Hansson et al. [16]	Post‐operative administration of 70 mg of Alendronate/weekly for 6 months after surgery	2 years	No significant difference in prosthesis migration between BP users and non‐BP users at 1‐year and 2‐year follow‐up (*p* > 0.05)	Post‐op BP treatment does not prevent early migration of TKA implants
Hilding et al. [21]	Peri‐operative administration of 1.6 g of Clodronate/daily for 3 weeks before and 6 months after surgery	1 year	BP users had reduced prosthetic migration compared to the non‐BP users (0.29 vs. 0.40 mm; *p* = 0.01)	Peri‐op BP treatment reduces the risk of early migration of TKA implants
Hilding et al. [20]	Peri‐operative administration of 1.6 g of Clodronate/daily for 3 weeks before and 6 months after surgery	4 years	BP users had reduced prosthetic migration compared to the non‐BP users on the transverse (*p* = 0.002) and vertical axis (*p* = 0.03)	Peri‐op BP treatment reduces the risk of migration of TKA implants
Hilding et al. [18]	Intra‐operative administration of 1 mg of Ibandronate on bone surface 1 minute before cementation	2 years	BP users had reduced prosthetic migration compared to the non‐BP users (0.32 vs. 0.45 mm; *p* = 0.006)	Intra‐op local BP treatment reduces the risk of early migration of TKA implants
Jaroma et al. [24]	Post‐operative administration of 10 mg of Alendronate/daily for 12 months after surgery	7 years	BP users showed significantly higher BMD in the femoral metaphysis at 4 years and in the lateral tibial metaphysis at 7 years compared to the non‐BP users (*p* = 0.024)	Post‐op BP treatment reduces periprosthetic TKA BMD loss
Katz et al. [25]	Peri‐op administration of bisphosphonates (starting minimum 12 months before surgery) Further details: NR	1 year (minimum)	All‐cause revision rate (any time): ‐ BP users: 1.89% ‐ non‐BP users: 1.90% (*p* = 1) Aseptic revision rate (1 year): ‐ BP users: 0.6% ‐ non‐BP users: 0.6% (*p* = 1) PPFx rate (1 year): ‐ BP users: 0.33% ‐ non‐BP user: 0.31% (*p* = 0.819)	Peri‐op BP treatment does not reduce the risk of aseptic revision and PPFx surgery at 1 year FU and does not reduce the risk of all‐cause revision surgery at any time
Lee et al. [28]	Peri‐op administration of bisphosphonates, dividing users based on duration of preoperative treatment into: ‐ LT: 3–5 years ‐ IT: 1–3 years ‐ ST: 0–1 years Further details: NR	2 years	PPFx rate: BP users: ‐ LT: 0.54% (90 days), 1.21% (2 years); ‐ IT: 0.54% (90 days), 1.15% (2 years); ‐ ST: 0.48% (90 days), 1.33% (2 years) non‐BP users: 0.43% (90 days), 1.01% (2 yrs) *p* > 0.05	Pre‐op BP treatment does not significantly reduce PPFx risk
Namba et al. [32]	BP users defined as: ‐ Patients treated for ≥6 months before revision surgery Or ‐ Patients treated for ≥6 months without undergoing revision surgery BP molecule: ‐ 91.7%: alendronate − 0.7% risedronate − 0.1% ibandronate − 8.3% > 1 BP	3.7 ± 2.7 years (mean)	All‐cause revision rate: ‐ BP users: 0.7% ‐ non‐BP users: 2.7% (*p* < 0.001) Aseptic revision rate: ‐ BP users: 0.5% ‐ non‐BP users: 1.6% (*p* < 0.001) PPFx: HR BP users/non‐users 3.78 (95% CI, 1.92–7.47; *p* < 0.001)	Peri‐op BP treatment reduces the risk of all‐cause revision, with exception of patients <65 yo with normal or no DEXA scan and the risk of aseptic revision, with exception of patients with normal DEXA scan. It also increases the risk of PPFx, especially in patients <65 yo with osteopenia
Ro et al. [38]	BP users defined as patients who had received at least 1 WHO‐defined daily dose of oral BP before revision surgery Further details: NR	14 years (up to)	Aseptic revision rate: ‐ BP users: 1.4% ‐ non‐BP users: 2.9% (*p* < 0.001)	Post‐op BP treatment reduces aseptic revision rates
Shih et al. [39]	Post‐operative administration of BP (Alendronate, Ibadronate, Risedronate, or Zoledronate) for at least 182 WHO defined daily doses Further details: NR	15 years (up to)	BP users had a lower risk of revision (*HR* 0.53; *p* < 0.001) and PPFx (*HR* 0.43; *p* < 0.001) than non‐BP users	Post‐op BP treatment reduces the risk of revision and PPFx following TKA
Soininvaara et al. [40]	Post‐operative administration of 10 mg of Alendronate/daily for 12 months after surgery	1 year	BP users had a lower periprosthetic BMD loss compared to non‐BP users (*p* < 0.015)	Post‐op BP treatment reduces periprosthetic BMD loss
Ueyama et al. [44]	Post‐operative administration of 35 mg of Alendronate/weekly until last observation	5 years	BP use was correlated with increased peri‐prosthetic BMD in the central femur (*r* = 0.39, *p* = 0.002), posterior femur (*r* = 0.39, *p* = 0.002), and medial tibia (*r* = 0.42, *p* = 0.007) No significant differences found in peri‐prosthetic BMD changes between mobile‐ and fixed‐bearing prostheses	Post‐op BP treatment increases peri‐prosthetic BMD, independently of the implant design
Wang et al. [47]	Post‐operative administration of 10 mg of Alendronate/daily for 6 months after surgery	3 years	BP users showed significant increases in peri‐prosthetic BMD compared with non‐BP users at six months (*p* < 0.01) and twelve months (*p* < 0.01). No significant difference in peri‐prosthetic BMD at thirty‐six months (*p* = 0.08)	Post‐op BP treatment increases peri‐prosthetic BMD in the first 12 months after TKA

Abbreviations: BMD, bone mineral density; BP, bisphosphonates; HR, hazard ratio; IT, intermediate‐term users; LT, long term users; PPFx, periprosthetic fractures; ST, short term users; TKA, total knee arthroplasty.

^a^ Total post‐TKA Follow‐up period unless otherwise specified.

The all‐cause revision rate was assessed in three studies [8, 25, 32] and was 1.5% (597/39812) for BP users and 2.3% (1376/60544) for non‐BP users. The aseptic revision rate was assessed in four studies [8, 25, 32, 38] and was 1.1% (1305/115731) and 2.5% (8030/316285) for BP users and non‐BP users, respectively. Moreover, Ro et al. [38] showed that the HR for aseptic revision was further reduced when the BP treatment period exceeded 1 year (*p* < 0.001).

The change in periprosthetic BMD from preoperative to postoperative assessment was reported in three studies [24, 40, 47], with a mean BMD variation of –0.04 in BP users and –0.2 in non‐BP users.

The implant migration, expressed as maximum total point motion (MTPM), was reported in four studies [16, 18, 19, 21], with values of 0.74 in BP users and 0.75 in non‐users.

The incidence of periprosthetic fractures was evaluated in four studies [8, 25, 28, 32], with a rate of 0.7% (369 events/50,388 patients) in BP users and rate of 0.5% (374 events/75,688 patients) in non‐BP users.

Overall, no substantial differences in outcome rates were observed between studies with short‐term and long‐term follow‐up.

**Figure 2:**
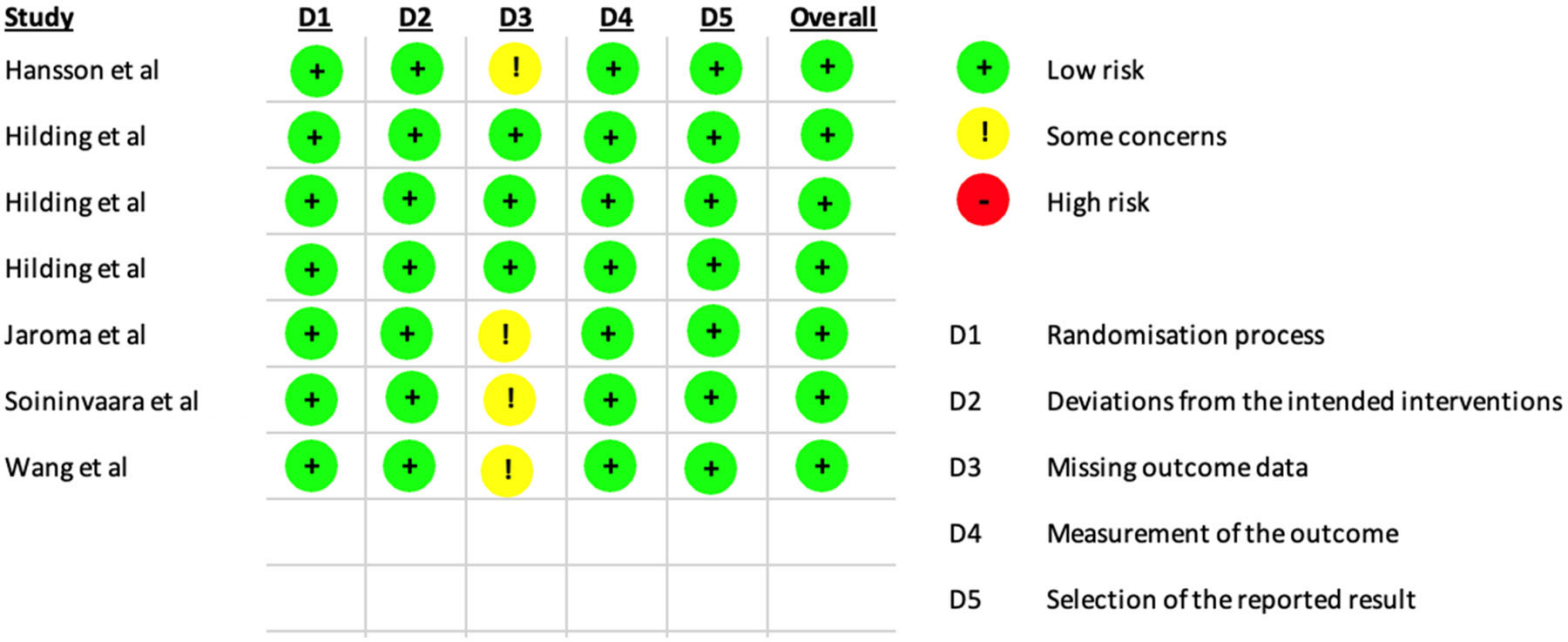
Risk of bias assessment of the randomised controlled trials (RCTs) using the ROB 2 tool.

**Figure 3:**
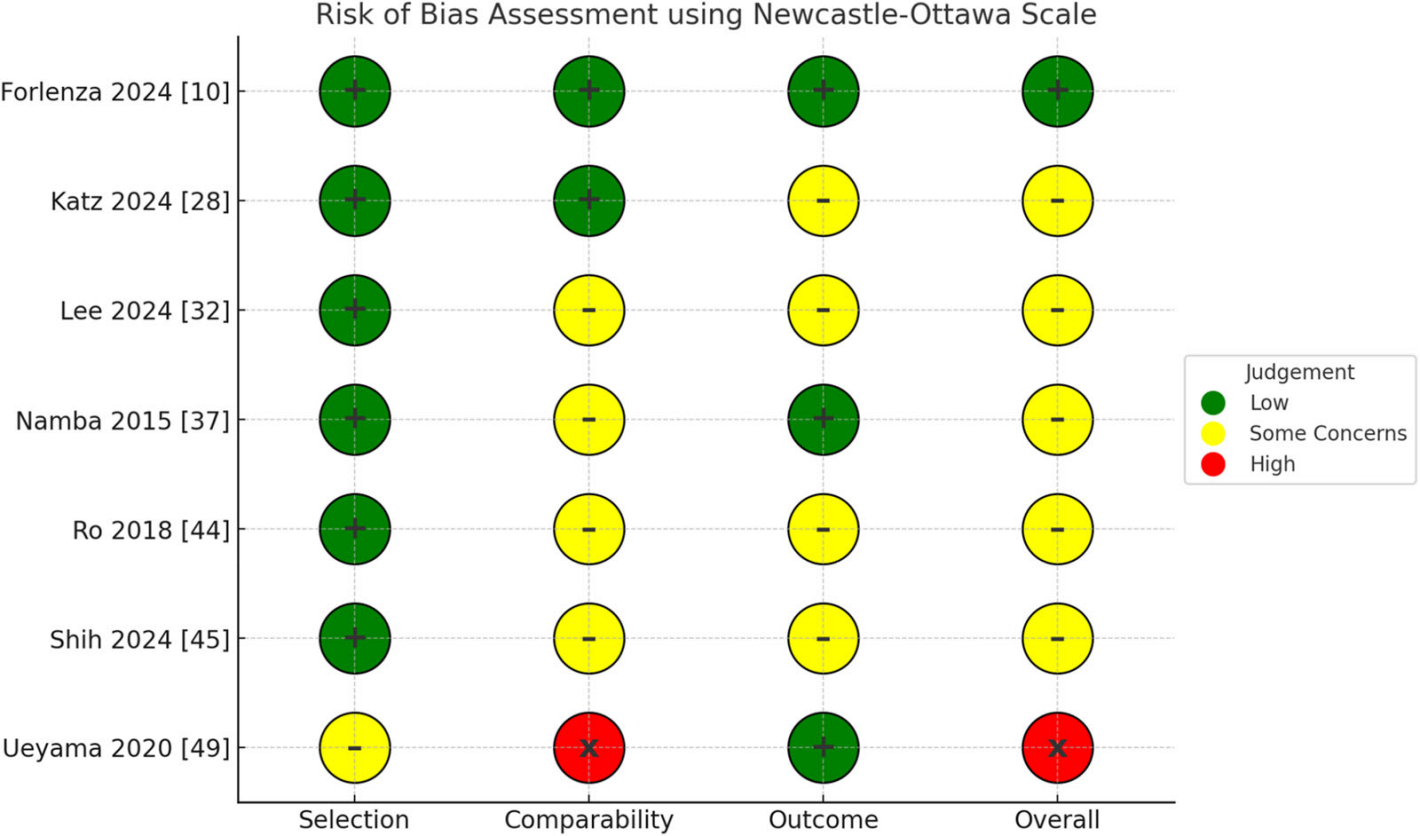
Quality assessment of the observational studies using the Newcastle–Ottawa Scale (NOS).

For the 7 RCTs included, the ROB 2 showed low risk of bias (Figure 2). For observational studies, the NOS identified one study with low risk of bias, five studies with some concerns, and one study with high risk of bias (Figure 3).

## DISCUSSION

The main finding of this study was that bisphosphonate therapy is associated with a reduced incidence of overall and aseptic revisions, as well as decreased periprosthetic BMD loss in patients undergoing total knee arthroplasty. To our knowledge, this is the first systematic review of the current literature on the topic.

Osteoporosis remains an under‐recognised issue among patients undergoing TKA, affecting nearly 25% of these patients [49]. The prevalence is even higher—nearly 39%—among postmenopausal women, due to the accelerated decline in bone density associated with hormonal changes [27, 49].

Existing literature has demonstrated that osteoporotic patients undergoing arthroplasty have a significantly increased risk of revision surgery and periprosthetic fractures [17, 37]. As osteoporosis and osteoarthritis frequently coexist in the elderly population [2, 14, 27], the role of BPs in TKA patients is becoming a subject of growing interest in clinical research. This is especially relevant considering that osteoporosis is associated with higher rates of 90‐day readmission, transfusion and surgical complications [31], and that up to 80% of osteoporotic TKA patients do not receive a specific pharmacological treatment [2, 27].

Recent studies suggest that BPs mitigate bone loss around prostheses by reducing bone turnover, stabilising BMD and improving stress distribution at the bone‐implant interface [1, 44] in the early postoperative period, during which significant bone resorption typically occurs [1, 30, 34]. Hilding et al. [18] also explored the effect of local application of ibandronate on the tibial bone cut, showing a significant reduction in implant migration as measured by radiostereometric analysis. The reported reduction in BMD loss around TKA implants is particularly relevant for alignment techniques—such as mechanical alignment—that do not respect the patient's preoperative phenotype [13], leading to altered loading patterns post‐surgery and to stress‐shielding phenomenon [9]. By countering the adaptive bone loss in unloaded areas, BPs could help maintain bone stock and prosthesis stability [29, 35, 42], enabling a better implant‐bone fixation [15, 24]. Such assumption is supported by our results, which showed a lower rate of overall and aseptic revisions among BP users. However, results of individual studies need to be critically interpreted. For instance, Lee et al. [28] and Forlenza et al. [8] did not observe a significant benefit of BPs on aseptic loosening at 1‐ and 2‐year follow‐up, respectively. In contrast, studies with longer follow‐up periods reported protective effects: Namba et al. [32] and Ro et al. [38] found odds ratios of 0.33 and 0.48, respectively, and Shih et al. [39] reported a hazard ratio of 0.53. Notably, in the study by Namba et al. [32], the mean time to aseptic revision was 2.7 years, thus suggesting that studies with shorter follow‐up periods may have underestimated the long‐term benefits of BP therapy. By extension, this observation could likely apply to other postoperative outcomes, including periprosthetic fractures.

The effect of BP treatment on periprosthetic fractures remains inconsistent in the literature [3, 8, 28, 39]. Shih et al. [39] demonstrated that postoperative BP use was associated with a significantly lower risk of PPFx (HR 0.43; *p* < 0.001) compared to non‐BP users. Conversely, Lee et al. [28], in a retrospective study using the PearlDrive database, did not find any statistically significant reduction PPFx, regardless of preoperative BP treatment duration. Interestingly, a subsequent study by Forlenza et al. [8] using the same database reported a protective effect of BP use on PPFx only in patients with cementless implants, with no significant benefit observed in cemented TKAs.

So far, orthopaedic surgeons managing osteoporotic TKA patients have primarily focused on the technical aspects of the procedure, including the cementation technique [45, 46], the use of stemmed versus stemless implants [7, 11, 50], fixation methods (cemented vs. cementless) [4, 6, 12], and implant design (cruciate‐retaining or posterior‐stabilised implants) [43]. Our study offers an additional insight on the potential positive impact of BP treatment, which may benefit both osteoporotic and non‐osteoporotic TKA patients. For instance, Namba et al. [32], reported a lower revision risk in patients older than 65 years, regardless of BMD. Additionally, the recently demonstrated anti‐biofilm properties of BPs [22] may help explain the findings of Shih et al. [39], who observed not only reduced revision rates but also decreased periprosthetic joint infection and all‐cause mortality in BP users.

### Limitations

This study has few limitations to be acknowledged. The included studies exhibited high heterogeneity in terms of design, follow‐up duration, demographic factors (e.g., patient BMD, physical activity and smoking), BP treatment regimens (e.g., molecule, dose, administration type, timing, duration and adherence to therapy) and intraoperative variables (e.g., surgical proficiency, implant type, stemmed vs. stemless implants, cementation technique, cemented vs. hybrid vs. cementless fixation). In addition, some studies reported outcomes as hazard ratios rather than raw event counts, and definitions of periprosthetic fractures varied across studies. For instance, Namba et al. [32] reported a hazard ratio of 3.78 for periprosthetic fractures in BP users, but included atypical proximal femoral fractures in their definition of PPFx, which may have inflated the risk estimate. These inconsistencies and data limitations prevented the execution of meta‐analyses for the outcomes of interest and underscore the need for well‐designed randomised clinical trials to validate our findings and guide clinical practice.

## CONCLUSIONS

Bisphosphonate use in patients undergoing total knee arthroplasty is associated with lower rates of overall and aseptic revisions, reduced periprosthetic BMD loss, and inconclusive effects on periprosthetic fractures and implant migration.

## AUTHOR CONTRIBUTIONS

Giancarlo Giurazza had the idea for the article and was responsible for writing of the manuscript. Stefano Campi and Edoardo Franceschetti were responsible for conceptualisation and supervised data acquisition and analysis. Marco Edoardo Cardinale and Francesco Rosario Parisi were responsible for data acquisition and extraction. Pietro Gregori was responsible for data analysis. Augusto Ferrini was responsible for realisation of Figures and Tables. Giuseppe Francesco Papalia qualified as corresponding author. Rocco Papalia and Michele Paciotti were responsible for reviewing and critically revise the manuscript. All authors have given final approval of the version to be published.

## CONFLICT OF INTEREST STATEMENT

The authors declare no conflicts of interest.

## ETHICS STATEMENT

The authors have nothing to report.

## PROSPERO REGISTRATION NUMBER

CRD420251004575.

## Data Availability

The data that support the findings of this study are available from the corresponding author, [G.F.P.], upon reasonable request.
